# A Research Agenda for Helminth Diseases of Humans: Health Research and Capacity Building in Disease-Endemic Countries for Helminthiases Control

**DOI:** 10.1371/journal.pntd.0001602

**Published:** 2012-04-24

**Authors:** Mike Y. Osei-Atweneboana, Sara Lustigman, Roger K. Prichard, Boakye A. Boatin, María-Gloria Basáñez

**Affiliations:** 1 Council for Scientific and Industrial Research, Water Research Institute, Department of Environmental Biology and Health, Accra, Ghana; 2 Laboratory of Molecular Parasitology, Lindsley F. Kimball Research Institute, New York Blood Center, New York, New York, United States of America; 3 Institute of Parasitology, McGill University, Montreal, Canada; 4 Lymphatic Filariasis Support Centre, Department of Parasitology, Noguchi Memorial Institute for Medical Research, University of Ghana, Legon, Ghana; 5 Department of Infectious Disease Epidemiology, School of Public Health, Faculty of Medicine (St Mary's campus), Imperial College London, London, United Kingdom; Swiss Tropical and Public Health Institute, Switzerland

## Abstract

Capacity building in health research generally, and helminthiasis research particularly, is pivotal to the implementation of the research and development agenda for the control and elimination of human helminthiases that has been proposed thematically in the preceding reviews of this collection. Since helminth infections affect human populations particularly in marginalised and low-income regions of the world, they belong to the group of poverty-related infectious diseases, and their alleviation through research, policy, and practice is a sine qua non condition for the achievement of the United Nations Millennium Development Goals. Current efforts supporting research capacity building specifically for the control of helminthiases have been devised and funded, almost in their entirety, by international donor agencies, major funding bodies, and academic institutions from the developed world, contributing to the creation of (not always equitable) North–South “partnerships”. There is an urgent need to shift this paradigm in disease-endemic countries (DECs) by refocusing political will, and harnessing unshakeable commitment by the countries' governments, towards health research and capacity building policies to ensure long-term investment in combating and sustaining the control and eventual elimination of infectious diseases of poverty. The Disease Reference Group on Helminth Infections (DRG4), established in 2009 by the Special Programme for Research and Training in Tropical Diseases (TDR), was given the mandate to review helminthiases research and identify research priorities and gaps. This paper discusses the challenges confronting capacity building for parasitic disease research in DECs, describes current capacity building strategies with particular reference to neglected tropical diseases and human helminthiases, and outlines recommendations to redress the balance of alliances and partnerships for health research between the developed countries of the “North” and the developing countries of the “South”. We argue that investing in South–South collaborative research policies and capacity is as important as their North–South counterparts and is essential for scaled-up and improved control of helminthic diseases and ultimately for regional elimination.

## Introduction

During their deliberations, and in the previous reports of this collection [1]–[7], the members of the Disease Reference Group on Helminth Infections (DRG4)—established in 2009 by the Special Programme for Research and Training in Tropical Diseases (TDR)—identified research gaps and priorities relevant to their thematic areas and contributed to the setting of a research agenda for the control and eventual elimination of human helminthiases. However, the group soon realised that the setting of a research agenda, although useful for advocacy and funding purposes, would not, by itself, fully contribute to the alleviation of the problem of helminthiasis [2]. The establishment and strengthening of research capacity in disease-endemic countries (DECs) are absolutely essential to make the control and elimination of these infections truly achievable and sustainable in the long-term. In the context of this paper, DECs are countries endemic for poverty-related infectious diseases in general and helminthiases in particular. Consequently, the issues concerning capacity building and research policies in DECs received the special attention of the DRG4 group after the Rio de Janeiro meeting in October 2010 [1]. In this paper, we focus on the research and development needs of these important aspects, especially in the context of Africa. We argue that investing in South–South collaborative research policies and capacity is as important as their North–South counterparts and is essential for scaled-up and improved control of helminthic diseases and ultimately for regional elimination.

## Inequalities in Research Capacity

Building research capacity is a long-term process that requires a systemic and inter-sectoral approach to developing appropriate regulatory frameworks (including the establishment of institutional review boards for ethical clearance of research proposals and research governance), building and maintaining physical infrastructure, and investing in human resources, equipment, and training in an environment conducive to research commitment and institutional support [8]. Above all, it requires demand and supply for enhanced scientific research, based on a conviction that research, and particularly health research, can improve the lives of people and spur economic development. In this paper, health research refers to an umbrella term encompassing research in biomedical, epidemiological, public health, social science, and environmental (among others) disciplines related to human health. The level of infectious and parasitic disease research capacity varies greatly across the world, and significant disparities exist in research and technological expertise and facilities between developed and developing countries. Substantial heterogeneity also exists within the latter—in Africa, for instance, South Africa (classified as “scientifically proficient”), and Benin, Egypt, and Mauritius (“scientifically developing”) have done reasonably well regarding national investment and productivity in science and technology, with the remaining countries in the continent falling behind (“scientifically lagging countries”) [9]. Inequalities in health research contribute to inequalities in health and ultimately wealth. Some countries, such as Brazil and the People's Republic of China, have made remarkable progress, in part because their governments have invested substantially in health research and capacity building. For science to deliver its promise of improving health and enabling development, all countries should be able to participate equitably in research [8].

Inter-country differences are mainly due to major investments that have been made by the developed world towards research and development (R&D) activities, especially in the proportion of the gross domestic product (GDP) that the countries' governments are willing to invest in research for an expected return. In some developed countries, long-term investment has resulted in extensive infrastructure, existence of national expertise and national and international academic prestige, a tradition in research funding, and a more expeditious path between basic and clinical, translational research and its implementation into public health policy and practice. Investments in research and innovative technologies have tremendously improved health in the developed world because of these countries' clear health research policies, including the setting up of priorities at institutional, national, and regional levels. The readily available and opportune deployment of such resources has led to rapid advances in controlling infectious agents that have epidemic potential. However, in the developing world, and especially in most African countries, adequate investments by most DECs in research capacity building to support prevention, control, and elimination of infectious diseases of poverty are insufficient. In addition to the paucity of highly trained researchers, there is a considerable brain drain of the already scarce numbers of trained professionals, fragmentation of research with much duplication of efforts, and a lack of focus on distinct national needs. According to Chauhan [10], lack of encouragement, unethical research practices that have left a legacy of mistrust, a colonial past that has left some degree of suspicion and engendered dependency, and most importantly, an environment of political, social, and economic instability, have all contributed to the scarcity of scientific research in Africa. Whatever the reasons, the dearth of research conducted in Africa for Africa is untenable [8] and threatens the long-term sustainability of any disease control programme.

The purpose of this review is to examine the level of capacity building in DECs, highlight some of the challenges that hinder the development of health research capacity with particular reference to poverty-related infectious diseases, summarise (not exhaustively) available research capacity building initiatives and policies and their implications for helminthiasis research and control, and provide recommendations for improvement of research capacity building towards the control and elimination of human helminthiases. Box 1 lists the abbreviations used in this paper, and Box 2 presents five salient points for capacity building in helminthiasis research.

**Box 1. List of Abbreviations**

**ACBF,** African Capacity Building Foundation

**APOC,** African Programme for Onchocerciasis Control

**AusAID,** Australian Agency for International Development

**BioMalPar,** Biology and Pathology of Malaria Parasite Network

**B&MGF,** Bill and Melinda Gates Foundation

**CAPES,** Brazilian Federal Agency for the Support and Evaluation of Graduate Education

**CDTI,** Community-Directed Treatment with Ivermectin

**CGMRC,** Centre of Geographical Medicine Research Coast

**CNPq,** National Research Council of Brazil

**CSRS,** Swiss Centre for Scientific Research, Côte d'Ivoire

**DBL,** Danish Bilaharziasis Laboratory-Institute for Health Research and Development

**DEC,** disease-endemic country

**DFID,** Department for International Development, United Kingdom

**DIMACS/MBI,** Center for Discrete Mathematics and Theoretical Computer Science/US–African Biomathematics Initiative

**DRG4,** Disease Reference Group on Helminth Infections

**EFINTD,** European Foundation Initiative for African Research into Neglected Tropical Diseases

**FIOCRUZ**, Oswaldo Cruz Foundation

**GDP,** gross domestic product

**HKI,** Helen Keller International

**ICEMR,** International Center of Excellence for Malaria Research

**IDRC,** International Development Research Centre, Canada

**IRD,** Institut de Recherche pour le Développement, France

**JAF,** Joint Action Forum (APOC)

**JICA,** Japan International Cooperation Agency

**KEMRI,** Kenya Medical Research Institute

**KFPE,** Commission for Research Partnerships with Developing Countries, Switzerland

**KNUST,** Kwame Nkrumah University of Science and Technology, Ghana

**MDG,** Millennium Development Goal

**M&E,** monitoring and evaluation

**MRC,** Medical Research Council, United Kingdom

**NGDO,** non-governmental development organisation

**NTD,** neglected tropical disease

**OCP,** Onchocerciasis Control Programme in West Africa

**PDP,** product development partnership

**RAPLOA,** rapid assessment procedure for loiasis

**R&D,** research and development

**REA,** rapid epidemiological assessment

**REMO,** rapid epidemiological mapping of onchocerciasis

**RNSA,** Regional Network for Schistosomiasis in Africa

**RNAS+,** Regional Network for Asian Schistosomiasis and other Zoonotic Helminths

**SCI,** Schistosomiasis Control Initiative

**SCORE,** Schistosomiasis Consortium for Operational Research and Evaluation

**SPHD,** Section of Parasitology, Health and Development (of former DBL)

**STH,** soil-transmitted helminthiasis

**Swiss TPH,** Swiss Tropical and Public Health Institute

**TB,** tuberculosis

**TDR,** Special Programme for Research and Training in Tropical Diseases

**TWAS,** The Academy of Sciences for the Developing World

**UNICEF,** United Nations Children's Fund (formerly United Nations International Children's Emergency Fund)

**UNDP,** United Nations Development Programme

**USAID,** United States Agency for International Development

**VBD,** vector-borne disease

**WHO,** World Health Organization

**Box 2. Summary Points for Capacity Building in Helminthiasis Research**

- There is great disparity in research capacity for parasitic diseases between the developed countries of the North and the developing countries of the South as well as among, and within, the latter. Inequalities in health research§ contribute to inequalities in health and ultimately wealth. These inequalities are even more pronounced in the case of infectious diseases of poverty and helminthiases
- There have been a number of high-level meetings on research for health in disease-endemic countries (DECs)‡, with the Bamako Call to Action 2008 concluding that to remedy the above, a greater proportion of the countries' GDP should be invested in science and technology and at least 2% of the ministries of health's budgets should be invested in research and research capacity
- Those countries of the South (e.g., Brazil, People's Republic of China, Cuba, India) that have invested substantially in biomedical research and research and development (R&D) have greatly increased their scientific output, halted or reversed brain drain, and excelled at product development partnerships and innovation (e.g., diagnostics, reagents, drugs, vaccines)
- Capacity building is a long-term, systemic, and inter-sectoral process, of which training of scientists is only a component. A more comprehensive approach requires physical infrastructure, appropriate equipment, conducive research environment, regulatory frameworks including the establishment of ethical review boards, attractive pay and working conditions, and substantial government support, including a competitive national research funding agency and monetary investment
- There are a number of international initiatives aiming to strengthen capacity building and establish interdisciplinary and multinational teams addressing infectious diseases of poverty in general and neglected tropical diseases (NTDs) and helminthiases in particular. Nearly all are funded by industrialised nations, accentuating North–South alignment. Although these initiatives are very welcome, they remain somewhat unbalanced

^§^An umbrella term referring in this paper to research in biomedical, public health, social science, and environmental (among others) disciplines related to human health.

^‡^In the context of this paper, DECs are countries endemic for poverty-related infectious diseases, including HIV, tuberculosis, malaria, and emerging, zoonotic, and neglected tropical diseases.

## Health Policy and Research Capacity in Disease-Endemic Countries and Calls for Action

Research must focus on national priorities and high disease burden conditions in DECs, with emphasis on evaluating interventions that aim to strengthen research capacity and health systems, and activities that translate knowledge into action and benefits to the local population [8]. In many countries of sub-Saharan Africa, Asia, Latin America, and the Caribbean, neglected tropical diseases (NTDs) in general, and helminthiasis and polyparasitism in particular, inflict a high disease burden [1]–[3], [5], [11]. Adequate research capacity for the management of helminthiases and other infectious diseases of poverty, including the NTDs, forms an essential component of the tools needed to meet the Millennium Development Goals (MDGs) [1]. Of particular importance is the demonstration of a measurable impact on health, educational success, and economic development, which are essential to convince government officials that financial investment in control programmes generates a tangible, cost-effective return [12].

To achieve the MDGs, several recent high-level meetings on research capacity and policy have called for action on health research (Mexico City in November 2004 [13], Abuja in May 2006, Accra in June 2006 [14], and Bamako in November 2008 [15]). From the statements made and undersigned in these meetings, it is clear that African governments have recognised the importance of adopting sound policies on health research and the potential positive implications such policies may have on the health and development of their nations. The policy issues on health research at both national and regional levels aimed at developing and strengthening adequate national health research policies and strategic frameworks based on national health research and knowledge systems, as well as strengthening existing, or creating novel South–South and South–North cooperation partnerships, including technology transfer and research capacity building [15]. In the Bamako Call to Action on Research for Health (2008) held by ministers and representatives of ministries of health, science and technology, education, foreign affairs, and international cooperation from 53 countries, the focus was on developing and strengthening policies on health research and innovation for health, and development of equity at national and regional levels [15]. All stakeholders were urged to “promote and share the discovery and development of, and access to, products and technologies addressing neglected and emerging diseases which disproportionately affect low- and middle-income countries”. Another important theme that ran through these meetings was the need for financial investment in health research by all African governments, including a pledge for the allocation of at least 2% of national health expenditure, and at least 5% of external aid, for health projects and programmes into research and research capacity building [16]. However, a clear commitment to meet the resolutions deriving from such meetings has not yet been made by all participating nations, particularly those of sub-Saharan Africa. In addition, it was recognised that “the nature of research and innovation for health improvement, especially in the context of the United Nations Millennium Development Goals, is not sufficiently inter-disciplinary and inter-sectoral; there is a need to mobilize all relevant sectors (public, private, civil society) to work together in effective and equitable partnerships to find needed solutions”.

It is, in our opinion, important that developing countries, supported by developed countries and donors, establish internally competitive national, or regional, research support and training agencies that can prioritise areas of national (regional) interest for potential support, be transparent and conduct open competitions for the best projects, scientifically and in terms of potential impact, have the possibility for a level of sustainability, integrate research and training, and help leverage external funding to support the national and regional efforts in research and training.

It is evident that more interaction among nations with similar health problems and common infectious diseases is essential to facilitate exchange of experiences as well as training of individuals to help achieve the MDGs. This requires a great deal of investment from both international and national funding bodies to develop the facilities and the capabilities of scientists who can drive research aimed at developing more effective tools and strategies to fight infectious diseases of poverty. Improving prevention and control strategies for NTDs will result in poverty alleviation and consequent achievement of the MDGs. However, this will require a sincere commitment, a governmental political resolve, and competitive and transparent mechanisms to use health research as a driver towards sustainable human resource development, economic growth, and poverty reduction. Collaborative research is surely one of the best means for strengthening such research capacity, and in general, it has been the case that scientists in DECs welcome collaboration with the more industrialised nations of the North as a vehicle for overcoming barriers to conducting research, obtaining training and funding, and promoting the exchange of ideas. Unfortunately, scientists of DECs seem less enthusiastic about collaboration between countries within their own continents and regions [8]. In part, this is because research funding opportunities for such South–South collaboration have been limited (but see below for a number of recent Brazil–Africa initiatives).

It is also true that most efforts towards health research and NTD capacity building in DECs have been made with the impulse of institutions based in industrialised countries. One of the major international organisations that has played an important role in building research capacity is the Special Programme for Research and Training in Tropical Diseases (TDR), based at the World Health Organization (WHO). The TDR has over the past 30 years sponsored the training of graduates from DECs at both master's and doctorate levels, notwithstanding specialist technological training, and further support (in the form of re-entry grants) to return to their own countries and establish productive research. The emphasis is on developing the research, management, and leadership capacities of DEC scientists and fostering research environments for long-term sustainability, quality processes, and strategic partnerships (http://apps.who.int/tdr/svc/grants/calls/grants-dec-investigators-2010). More recently, in 2010, the TDR has sponsored research and training exchanges between African scientists from Niger, Nigeria, and Uganda and the National Institute of Parasitic Diseases, Chinese Center for Disease Control and Prevention in Shanghai regarding schistosomiasis control. This has resulted in a fruitful South–South connection between two previously separate TDR-supported networks in schistosomiasis, namely, the Regional Network for Asian Schistosomiasis and other Zoonotic Helminths (RNAS+, http://www.rnas.org.cn) and the Regional Network for Schistosomiasis in Africa (RNSA, http://www.rnsa.org.zm). Through this newly cemented collaboration, the two networks can learn from one another to build their capacity and expertise (http://www.who.int/tdr/publications/documents/tdrnews86.pdf). Another significant step in the right direction is the proposed relocation to Africa of the TDR-supported Initiative to Strengthen Health Research Capacity in Africa (ISHReCA), at present based at TDR/WHO in Geneva, and sponsored by the Wellcome Trust, among other funders (http://ishreca.org/).

**Figure 1:**
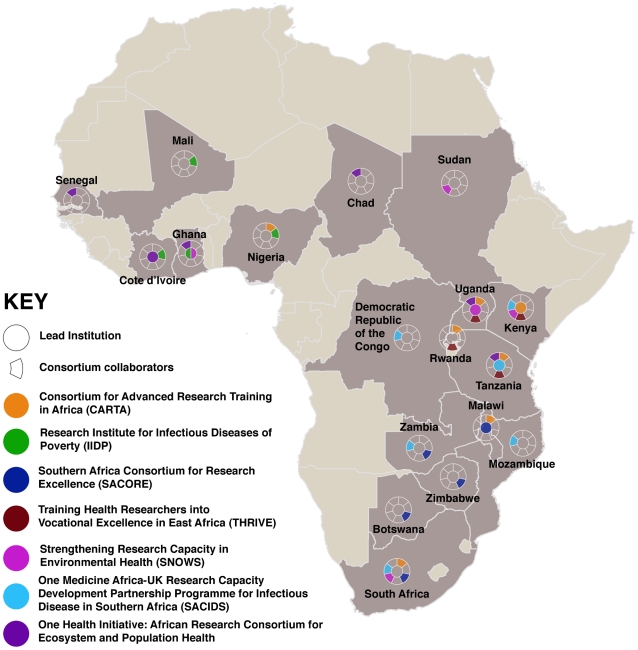
Countries and consortia in the African Institutions Initiative of The Wellcome Trust. (Redrawn from http://www.wellcome.ac.uk/news/2009/features/wtx055738.htm.)

In 2009, the Wellcome Trust funded the African Institutions Initiative, aiming to develop institutional capacity to support and conduct health-related research vital to enhancing people's health, lives, and livelihoods through the formation of seven new international and pan-African consortia (Figure 1), with each partnership being led by an African institution (http://www.wellcome.ac.uk/News/Media-office/Press-releases/2009/WTX055742.htm). This is in addition to longer-established African-based programmes such as the KEMRI-Wellcome Trust Research Programme at the Centre of Geographical Medicine Research Coast (CGMRC), in Kilifi, Kenya (http://www.kemri-wellcome.org/). There are many other such programmes, and Table S1 lists some examples of current capacity building initiatives in the area of health research and NTDs, particularly in Africa, with external support. The African Capacity Building Foundation (ACBF), though not focused on health, aims at building sustainable human and institutional capacity for poverty reduction in Africa. Since its inception in 1991, the ACBF has supported a total of 246 programmes and projects in some 44 sub-Saharan African countries and committed more than US$400 million to capacity building (http://www.acbf-pact.org/).

Regarding South–South initiatives, Brazil has, since 2008, supported collaboration and training of African scientists through the Pro-Africa Program for Thematic Cooperation in Science and Technology of the National Research Council (CNPq, http://www.cnpq.br). This scheme funds meetings, research, and seed money to evaluate potential on collaborative efforts. In partnership with the Academy of Sciences for the Developing World (TWAS, http://www.twas.org/), an autonomous international organisation based in Italy that promotes scientific capacity and excellence for sustainable development in the South, CNPq also supports students from African countries to be trained in Brazil at post-graduate and post-doctoral levels. Furthermore, the Oswaldo Cruz Foundation (FIOCRUZ), with support from the Brazilian government, recently established an initiative with the Mozambican National Institute of Health to create a master's programme in health sciences, with the goal of providing qualified human resources for health research and innovation for Mozambique [17]. For a more comprehensive account of Brazil's conception of South–South cooperation in health, the reader is referred to [18].

## Challenges for Research Capacity Building in Disease-Endemic Countries

As mentioned in the introductory paper of this collection [1], R&D investment in the areas of NTDs in general, and helminthiases in particular, pales into insignificance [11] in comparison to that made for HIV/AIDS, malaria, and tuberculosis (TB) according to the G-FINDER reports of 2009 and 2010 [19], [20]. It is, therefore, not surprising, given the overall shrinking levels of R&D investment made on helminthiases by industrialised nations [11], that in most DECs, research on NTDs and helminthiases is not considered a priority, receiving very little attention and being further hindered by some of the obstacles described below.

### Outflow of Trained Staff

The science and health sectors in Africa, and to a lesser extent Latin America and parts of Asia, suffer from a continuous outward drain of trained staff, a problem that donors have addressed primarily by financing training. But training is only part of the solution to building human capacity, because low salaries, poor and unattractive working conditions and environments, and lack of institutional incentives to allow the development of the individuals' full scientific potential in their own countries also contribute to low morale and high outflow. Technical assistance and training have often proved ineffective in helping to build sustained capacity. What is needed is a comprehensive approach to human resource management as well as a systemic approach to capacity building [21], including recognition of the importance of developing a strong research culture in DECs.

### Lack of Governmental Support

A report commissioned by the World Bank showed that with the exception of South Africa and Egypt and a few others, most DECs in Africa (and also in Latin America, with the exception of Brazil and Cuba, and Asia, excepting the People's Republic of China and India) incur very low national investments in research in general, and have a low productivity in science and technology [9]. This generalisation probably masks the fact that scientifically less advanced countries may have reasonable capacity in certain areas, but there is no doubt that the situation of health research in Africa is dire [8]. Another World Bank report [21] found that most capacity support remains fragmented, that the countries do not fully “own” the capacity building agenda, and that the challenges of capacity building vary across sectors within countries as well as across countries. Research capacity building requires the investment of meaningful amounts of funds, which many of the DEC governments are not willing to make because research is considered a long-term undertaking that only rich countries can afford, and because of other perceived pressing needs. Governments are inclined to follow agendas demanded by powerful, well-organised lobby interests more readily than those sought by seemingly weaker or more diffuse, decentralised interests, such as investment in education and health [21]. There is a lack of sufficient funds from the individual governments of DECs to support institutional infrastructure, to fund medium- and long-term research projects, and to create well-remunerated job opportunities for local scientists. Moreover, these are major factors preventing African scholars trained abroad from returning to their home countries to pursue careers in health research. Despite these small national inputs, research capacity development has been identified as an important endeavour that should be fostered in order to obtain the evidence-based knowledge that is relevant to the health concerns of local communities and that policy-makers can use for implementation of adequate practices [22].

### Underdeveloped Collaborations and Networks

Establishing and nurturing collaborations in research is undoubtedly one of the best vehicles for building and strengthening research capacity in DECs, and emphasis should be placed on long-term partnerships. Efforts to incorporate as co-workers multinational members of interdisciplinary teams can be of immense value, which should be encouraged and cultivated [10]. Regrettably, the collaborative links between the North and the South are stronger than those between South and South, probably because it is perceived that countries in the North can bring resources and technologies not available in the South. Feeble South–South networking prevents exchange of expertise between countries affected by the same infections and often sharing the same transmission zones, and imperceptibly contributes to the drain of the best researchers in the South towards the North. There is a need to strengthen South–South collaborations and to fund and establish research networks that support, for example, technology transfer aimed at the development and manufacturing of new diagnostic tools and anthelmintics for the management and control of helminthiases.

### Funding Issues and Ownership of Programmes

**Figure 2:**
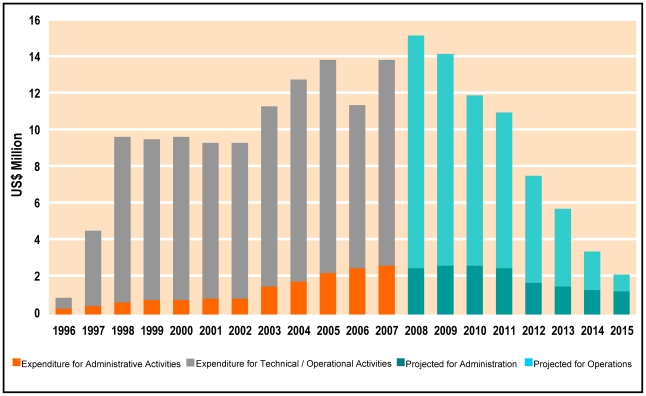
African Programme for Onchocerciasis Control Trust Fund annual expenditure. Since the inception of APOC in 1995, expenditure has increased steadily until 2007, mainly with external donor support (listed in http://www.who.int/apoc/about/funding/en/index.html). The orange and dark turquoise portion of the bars represents investment in administration, and the grey and light turquoise portions indicate support for technical activities, operations research, and capacity building. External funding has decreased since 2007 and it is anticipated that from 2015 onwards the programme will be fully devolved to participating countries and their NGDOs. Recently, APOC's mandate has been extended to 2025.

Most research capacity building efforts within and between DECs are supported entirely by external donor agencies, research funding bodies and foundations, pharmaceutical companies, and non-governmental development organisations (NGDOs) such as the World Bank (http://www.worldbank.org/); the Bill & Melinda Gates Foundation (B&MGF, http://www.gatesfoundation.org/); the Carter Center (http://www.cartercenter.org/index.html); Fogarty International Center (http://www.fic.nih.gov/); GlaxoSmithKline (http://www.gsk.com/); Helen Keller International (HKI, http://www.hki.org/); the Japan International Cooperation Agency (JICA, http://www.jica.go.jp/; see Figure S1) and others mentioned in Table S1 and below. For instance, the B&MGF, through a five-year grant to the University of Georgia Research Foundation, is funding the Schistosomiasis Consortium for Operational Research and Evaluation (SCORE), established in December 2008 to answer strategic questions about schistosomiasis control and elimination (http://score.uga.edu/). The challenge is how to ensure that low-income countries create a stable demand for sustainable control, anthelmintic distribution, and the research that supports these activities. In responding to this demand, it is crucial that there is an appropriate supply of trained personnel at various levels, and that the countries are able to raise adequate resources to complement or eventually replace what is already received from external sources, thus sustaining the success of control and elimination efforts made once donor fatigue occurs, or donor funding is diverted somewhere else. An excellent example of such a transition from external donor funding to internal, DEC funding is the African Programme for Onchocerciasis Control (APOC). APOC is funded entirely from voluntary contributions channeled through the APOC Trust Fund, and has fostered North–South, North–South–South, and South–South partnerships (Box 3 and Figure 2).

**Box 3. Research Capacity and the African Programme for Onchocerciasis Control**

The World Bank is the fiscal agent of the African Programme for Onchocerciasis Control (APOC); it manages the APOC Trust Fund and reports annually to the Joint Action Forum (JAF) on the financial situation of APOC. Community-directed treatment with ivermectin (CDTI) activities are funded through three mechanisms: trust funds available through APOC, contributions from the national governments of APOC countries, and funds from non-governmental development organisations (NGDOs). To extend APOC activities until 2015, US$60 million were raised during the JAF meeting in Brussels in December 2007, when in celebration of the 20th anniversary of the donation of Mectizan (ivermectin), the manufacturer and donor of the drug, Merck & Co., Inc., also became a financial donor of APOC.

Over 80% of funds are spent on technical and operational activities in endemic countries. Figure 2 shows how spending will decrease from 2008 to 2015, when APOC's mandate was intended to end. By this time, it is planned that funding for sustained CDTI will be provided fully by the governments and NGDOs of the participating countries, completing the transition from external to internal funding (http://www.who.int/apoc/about/funding/en/index.html).

Not only are the treatment activities of CDTI financed through this mechanism, but also APOC provides funds to encourage operations research and enable evaluation of its impact by multidisciplinary and international teams with a strong cadre of African scientists. Examples are the studies for the feasibility of elimination through insecticidal larviciding of the Bioko form of *Simulium yahense*, which have led to the elimination of this vector species on the Bioko island of Equatorial Guinea [47], and quantification of the impact of CDTI on skin disease [48].

APOC is an example of North–South support being extended to South–South cooperation. APOC and TDR have supported relevant operations research in the areas of rapid epidemiological mapping of onchocerciasis (REMO), rapid epidemiological assessment (REA) of onchocerciasis, rapid assessment procedure for loiasis (RAPLOA), and CDTI as a community-empowering strategy. All this has contributed significantly to improved capability and increased number of researchers in onchocerciasis-endemic countries of Africa. APOC has supported research in the onchocerciasis-endemic countries either financially (Figure 2) or by making high-level research experts from DECs or from other countries available as mentors for local researchers.

### Basic and Operations Research and Specialised Training

The successes achieved by control programmes, like the former Onchocerciasis Control Programme in West Africa (OCP), the current APOC, and the Schistosomiasis Control Initiative (SCI), have been partly realised because of the fundamental and operations research carried out within the umbrella of the programmes activities [1]. In most DECs, there is moderate capacity for research in the areas of epidemiology, parasitology, malacology, and entomology, but research capacity is lacking or dwindling in some other specialised areas that are essential to support successful control measures, such as transmission dynamics modelling (see DIMACS/MBI in Table S1), advanced statistical analysis of helminth and NTD epidemiological data, parasite population biology and genetics, vector ecology, and expertise for detection and monitoring of resistance to anti-parasitic drugs and anti-vectorial measures. Expertise in these areas and evidence-based research output are essential for supporting appropriate decisions by policy-makers in the context of implementation and evaluation over time of single and/or integrated helminth control programmes [6], [11]. In this context, authors of this paper (MYO-A, RKP, M-GB) recently organised and taught a course, funded by the Leverhulme–Royal Society Africa Award (Table S1), on “Epidemiology, Transmission Dynamics and Control of Vector-Borne (VBDs) and Neglected Tropical Diseases (NTDs)” at the University of Ghana, including topics on anthelmintic resistance, vector biology, infectious disease modelling, and NTD epidemiology and control.

### Insufficient Mentorship

In many DECs, scientists and/or lecturers from research institutions and universities work mainly as individuals rather than as teams. This leaves the new entrant, the young scientist/lecturer, in a place where there is little or no guidance, direction, or an environment that will facilitate his/her career development and progression. Moreover, facilities and inputs for scientific research available to the young scientist are limited. To facilitate research capacity in DECs, there is a need for senior scientists/faculty staff members at higher levels to mentor junior researchers. Having a mentor with expertise, peer esteem, and networking skills provides an invaluable triad function: guidance and direction for the development of a career pathway; research facilities, laboratories, and group support for practical experience; and access to a network of contacts for projection in the national and international arenas. Mentors and role models provide not only exposure to robust and demanding academic and research environments and a vision of what is expected and possible to achieve, but also opportunities to participate and present in international conferences, and obtain feedback on research dissemination activities, manuscripts, oral presentations, and grant preparation. The introduction of the junior fellowships by the European Foundation Initiative for African Research into Neglected Tropical Diseases (EFINTDs) (Table S1) is a step in the right direction, as young scientists will receive mentorship from experienced scientists both from strong research institutions in the North and in the South. This should help raise a generation of young scientists with the required expertise to themselves serve as mentors for the next generation. Mentorship programmes are crucial in DECs, since unlike the institutions of the developed nations, most DECs do not have the period of post-doctoral internship and further training that new PhD graduates often have access to in the North. It will be very helpful to DECs if international funding agencies were able to provide more fellowships with mentorship options for promising junior scientists, just after completing their PhDs, and who are willing to remain in or return to their home countries to build their own career with the intention of focusing in their country's research needs [23].

### Access to Literature and Unedited Databases

Strict open-access publication policies, subsidies by the research funders of wealthier nations, and the growth of prestigious and high impact open-access journals (for instance in the Public Library of Science and BioMed Central families) have ameliorated the access of scientists in DECs to high quality and updated peer-reviewed research. In particular, BioMed Central has recently launched “Open Access Africa”, a collection of initiatives designed to increase the output and visibility of scientific research published by African learning institutes. The Kwame Nkrumah University of Science and Technology (KNUST) in Kumasi, Ghana, is the first African Foundation Member to participate in BioMed Central's free membership scheme (http://www.biomedcentral.com/developingcountries/events/openaccessafrica). However, a considerable amount of investment by higher education and research institutions is needed to maintain the necessary funding for electronic journals, digitised archives, bibliographic databases, and printed literature that are not easily available in DECs. Furthermore, fast and reliable Internet access is sometimes lacking in DECs, which in turn limits the access of researchers in those countries to open-access information via the Internet. For a compilation of web-based bibliography databases of epidemiology, parasitology, and tropical medicine resources from the Spanish-speaking Latin America and Caribbean regions, see [24]. Without affiliation to a strong library in an academic institution, individual Internet access even at broadband speed is not sufficient. This hampers research and research capacity. As a positive example, the Brazilian Federal Agency for the Support and Evaluation of Graduate Education (CAPES) subscribes to all major peer-review journals, a remarkable effort to make scientific publications available to all academic and educational institutions in Brazil. In addition, CAPES works with the Cambridge Overseas Trust to offer the CAPES Cambridge Scholarship, which welcomed its first recipients (Brazilian nationals) for PhD training in October 2011 (http://www.cambridgetrusts.org/partners/capes-brazil.html).

The issue of open access to helminth epidemiology databases for the purposes of mathematical modelling is more fully discussed in [6]. This ongoing issue has been an important hurdle in developing collaborative programmes and has been addressed in the preparatory meetings for the Bamako Call to Action [25]. While several institutions would like to have all data collected from countries where diseases of the poor are prevalent placed on open-access databases, this proposal has not had general acceptance because it appears to give countries with highly structured, efficient institutions, access to data at apparently no cost, which have been generated, at great cost, by low- and middle-income countries. The analysis of these data should be conducted and shared between the latter before it is open to the former, or password-protected access could be granted after mutually beneficial agreements or memoranda of understanding for joint analysis and publication of hard-earned data have been signed by participant institutions and researchers [25]. There are also intellectual property issues needing further discussion [26], [27]. Investment in data collection and curation can be substantial, and there has been little mutual collaboration between the developed countries of the North using data from developing countries of the South. It is clear that capacity building for data analysis and translation of findings for improving health, together with new research questions to be addressed, need further discussion. A system needs to be developed where different stakeholders will participate and share findings [25].

### Lack of Advanced Enabling Technology Tools

DECs face challenges in research capacity for helminthiases and other infectious diseases, especially in areas that require the application of advanced technology for disease control such as functional genomics and bioinformatics research. This may be either due to the lack of trained personnel in such specialised areas, the lack of appropriate infrastructure and equipment, or the brain drain of the few local scientists and health professionals who may have such expertise. Research capacity building in DECs faces the greatest challenge in areas requiring the application of advanced technologies such as genetics for disease control, genomics, functional genomics (and other “omics”), bioinformatics, and computational biology that can, in the medium and long term, have a major impact on disease control or elimination [28], [29]. There is a lack of expertise in DECs for the development of new reagents, products and approaches for diagnosis, anthelmintics, vaccines, and integrated vector control, which are crucial for the sustained success of current programmes for the control and elimination of helminthiases [30]. If DECs had the adequate research capacity for the development of effective functional genomics tools and bioinformatics, areas like the study of gene function could be applied for the development of novel drugs based, for instance, on locally available natural products (see the LANBIO and ANDI initiatives described in Text S1).

The path forward is not impossible, however. Some DECs in South America, the Caribbean, and the African regions, despite similar challenges, have been able to develop adequate research capacity. For example, Brazil, Cuba, and South Africa have made major technological advancements in the field of functional genomics, bioinformatics, and vaccine development. Notably, the scientific output and impact of these countries' researchers have increased internationally, and consequently the brain drain has been reduced or halted [31]. Such progress is mostly due to the financial investments made by the governments of such countries to build and support adequate research capacity in their national institutions. This is now yielding expertise in new technologies, leading to the development of innovative interventions and effective management of various diseases, with resultant progress in infectious disease control. For instance, the first effective meningitis B vaccine was developed at the Cuban Finlay Institute (http://www.finlay.sld.cu/english/eindex.htm), and was recently licensed to GlaxoSmithKline [32]. The FIOCRUZ/Bio-Manguinhos and Butantan Institutes of Brazil and other collaborative institutions are full members in the product development partnerships (PDPs) for the Human Hookworm Vaccine Initiative [33]. The People's Republic of China is one of the world's leading producers of penicillin, and together with India and Brazil, produces praziquantel for schistosomiasis treatment. The National Institute of Parasitic Diseases, Chinese Center for Disease Control and Prevention in Shanghai, following a collaborative effort between Chinese, European, and African scientists, has investigated the effects of artemether, singly or in combination with praziquentel, against the major human schistosome species [34]. The Serum Institute of India is the world's leading manufacturer of diphtheria-pertussis-tetanus vaccine. Over 60% of the United Nations Children Fund's vaccine requirements for the Expanded Programme on Immunization are met by Brazil, Cuba, India, and Indonesia [35].

### Scarce Evaluation of Capacity Building and Research Partnership Coalitions

A further challenge hampering the effective development of research capacity building is the lack of application of the same rigorous monitoring and evaluation (M&E) practices to capacity building work that are implemented in other areas, with most activities lacking standard quality assurance processes at the design stage, and not being routinely tracked, monitored, and evaluated [21]. Scientific training and outputs could, for instance, be measured in terms of abundance and quality of research dissemination activities; numbers of peer-reviewed papers published by authors from DECs; number of grants in which the principal investigator and research instigator are based in DECs; number of trainees at undergraduate and post-graduate levels who receive education in DECs or return to their home countries to pursue health research careers; and number and impact of courses, workshops, internships, or academic visits hosted or organised in DECs, among others. Sustainability outputs could be assessed in terms of having created a demand for sustainable parasite control and for trained, local personnel to conduct and supervise it as well as a corresponding supply and retention of such personnel; impact of research findings on policy and practice, and in terms of the countries' capacity to assume ownership of and co-finance control programmes implementation and M&E [23], [36].

## National, Regional, and Global Efforts and Strategies towards Capacity Building for Research in Infectious Diseases of Poverty

### North–South Partnerships

Given the lack of political will and financial commitment by most DECs to support research capacity building, the role of global and regional efforts has become crucial in supporting and sustaining the control of helminth infections. These efforts include various established research partnerships between the developed countries and the developing nations. Establishing these North–South partnerships in the form of consortia, networks, and collaborations between research institutions has made valuable contributions to research capacity and should be encouraged, although this requires significant financial investments [31], [37], [38]. These partnerships are essential for the training of skilled personnel in research methods and dissemination, the translation of the results of research into tangible actions, products, or improved practices and policies for the benefit of communities and individuals [31], [39], [40], and the deployment of current interventions or the development of novel strategies within national, regional, and global control programmes [38]. Text S1 describes examples of such partnerships for the research and control of helminthiases, the investigation of infectious diseases of poverty, poverty elimination, and environmental sustainability (see also Figure S1). For a more comprehensive and general account of international initiatives for building research capacity in the South, the reader is referred to [41].

For such partnerships to work effectively, they should include major players such as local research institutions, universities, and researchers on infectious diseases of poverty, managers of control programmes, and policy-makers. They should also provide a forum for an active involvement of the DECs and their scientists to ensure that the priority needs of these countries, as well as the training of local human resources, are met. For a more comprehensive account of “desirables” in establishing “win-win” partnerships between the North and the South, readers are referred to the “11 Principles for Research in Partnership with Developing Countries” prepared and published by the Swiss Commission for Research Partnerships with Developing Countries (KFPE) [42], and more recently extended to 12 principles [43], [44]. Bonfoh et al. [40] discuss how the application of these principles, and the evolution of the partnerships from a very basic field station, driven by external projects, to a fully fleshed research centre, partnered with other African institutions, have ensured that research at the Swiss Centre for Scientific Research (CSRS) in Côte d'Ivoire has survived a decade of serious civil unrest.

Malaria research initiatives are good examples of such integrated successes. Although it is a large and highly competitive field, a number of networks exist to foster collaboration, communication, and interactions not only amongst international members, but also among local members. An example is the Biology and Pathology of Malaria Parasite (BioMalPar), a network of excellence funded by the European Commission, which has been successful in establishing and strengthening malaria communities and laboratories in both Europe and malaria-endemic countries [45]. A more recent example, described in Text S1, is the creation of International Centers of Excellence for Malaria Research (ICEMR), which has established a global network of independent research centres in malaria-endemic settings to provide knowledge, tools, and evidence-based strategies to support researchers working in a variety of endemic areas, especially within governments and health care institutions.

### South–South Partnerships

In addition to these North–South partnerships, research capacity building can be reinforced by facilitating and providing more opportunities for South–South collaborations [17], [18]. Text S1 also describes some of these initiatives. For instance, in the Latin American and Caribbean region, Brazil and Cuba have made major research investments resulting in a calibre of research expertise and research institutions that are recognised internationally [18], [32]. These well-established institutions could play a major role as regional and inter-continental focal points for South–South collaborations and capacity strengthening for other endemic countries such as the Brazil–Africa programmes previously described [17], [18]. In Africa, only a few countries, such as South Africa, have developed sound fiscal policies supporting knowledge-based development and leading to wealth creation. This has enabled them to invest in science and technology, build substantial research capacity [9], and importantly, to provide attractive remuneration packages to keep their scientists and other expertise in the country. With such expertise and infrastructure, South Africa could also serve as a regional focal point for South–South collaborations within Africa and between other DECs.

Since such partnerships involve considerable financial commitment, extended and continued support will still be needed from global and regional donors, including the WHO, TDR, and other major agencies, schemes, research and development institutions, and funding bodies committed to capacity building such as the Health Programme of the European Commission (http://ec.europa.eu/health/programme/funding_schemes/index_en.htm); the training and capacity building programmes of the Section for Parasitology, Health and Development (SPHD, http://www.ivs.life.ku.dk/English/Sections/SPHD.aspx) of the former Danish Bilharziasis Laboratory (DBL–Centre for Health Research and Development), and of the former Swiss Tropical Institute (now Swiss Tropical and Public Health Institute [Swiss TPH)] in Basel, http://www.swisstph.ch/); the US Agency for International Development (USAID, http://www.usaid.gov/), and in particular the USAID's Neglected Tropical Disease Program (http://www.neglecteddiseases.gov/index.html); the Neglected and Other Infectious Diseases Program of the B&MGF (http://www.gatesfoundation.org/topics/Pages/neglected-diseases.aspx); the New York-based Ford Foundation International Fellowships Program (http://www.fordifp.net/); the International Development Research Centre of Canada (IDRC, http://www.idrc.ca); AusAID (http://www.ausaid.gov.au/) in Australia; the Institut de Recherche pour le Développement (IRD, http://www.ird.fr/) in France; the Department for International Development of the United Kingdom (DFID, http://www.dfid.gov.uk/); the Wellcome Trust (http://www.wellcome.ac.uk/), and the Medical Research Council (MRC, http://www.mrc.ac.uk/index.htm) also in the UK, as well as other foundations, initiatives, and programmes (such as those listed in Table S1, summarised in Text S1, and described in [41], which also provide awards to support candidates from DECs for post-graduate studies at master's and doctoral levels, post-doctoral careers, and research projects). However, these funding opportunities are highly competitive, and therefore for nationals of DECs to access such funds, local scientists should establish strong networks and collaborations and also strengthen their publication and proposal-writing skills to enable them to tap into such opportunities for research capacity building [23].

An important need to be addressed by both North–South and South–South partnerships is that of improving the graduate-level training for students in DECs. Unfortunately, the NTD knowledge base in DECs is often not extensive and the investigators who are involved in intervention programmes are usually associated with ministries of health rather than with national universities. Although this still would entail a great deal of involvement by partners from the North, if external universities could provide support for investigators to provide in-country training within DECs to build up a critical mass of able personnel, the ability to carry out and evaluate NTD control without relying on external direction will one day become a reality. Often, for good training, DEC students must travel elsewhere, which contributes to the exit of qualified scientists who stay in their country of training rather than returning to their home countries (but see Text S1 and Table S1 for examples of current initiatives aiming to remedy this very problem).

## Concluding Remarks and Recommendations

Support for and from DECs for building relevant, DEC-led research capacity for the control and elimination of human helminthiases and infectious diseases of poverty is still inadequate despite the many initiatives that exist, as these initiatives mainly focus on research portfolios, researcher profiles, and administrative requirements of the developed world [23], [46]. Thus, a large proportion of the funding for building research capacity in DECs has tended to originate from donor agencies, funding bodies, and research institutions whose epicentre is not located in DECs, and whose principal investigators and research leaders represent the interests of academic centres from the North rather than those from the South. There is a vast disparity in the capacity to conduct world-class research between the nations of the North and the less developed countries of the South, partly based on a lack of understanding of the potential for scientific research in general, and health-associated research in particular to give the countries' economies and development the necessary knowledge base to break out of the poverty cycle. However, this potential will not be realised if research does not translate into policies, actions, and products destined to improve the situation of the afflicted populations and break the cycle of poverty and deprivation that the NTDs inflict on the most marginalised populations of the world. This requires a concerted effort by the DECs and the many national, regional, and global initiatives towards the development of true, win-win partnerships. This would be a welcome path towards meeting the MDGs.

In addition to the dearth of capacity in operations research and appropriate, low-cost technologies to support the implementation and, importantly, the M&E of helminthiases control programmes by DECs, these countries also face challenges in areas that require the application of advanced technology tools for disease control such as genetics methods for the control of parasites and their vectors, functional genomics, bioinformatics, computational biology, and the development of new and optimised products for diagnosis, treatment, and prophylaxis. This technological gap further accentuates the chasm between the North and the South and contributes to deepen the sense of dependency and inferiority that prevents the DECs from fully assuming the responsibility of tackling their pressing health needs and owning the intervention programmes. Other areas of importance for the confident development of research, which is appropriate and flexible in light of ongoing interventions, is the lack of training in transmission dynamics, epidemiological modelling [6], the prompt and opportune detection of resistance to anti-parasitic drugs and anti-vectorial products [3], and the economic evaluation of interventions for demonstrating their cost-effectiveness [12] to the scientific, donor, and political communities. Some policies on health research and capacity building have already been developed and implemented at both national and regional levels in the Africa, Asia, Latin America and the Caribbean, and the Pacific island regions. However, more concerted efforts are required to ensure that the gaps in research capacity, commitment, and R&D investment become narrower rather than wider in the current climate of global financial instability and constraint [20].

Policies supporting the development of effective and truly collaborative linkages and partnerships with international health research agencies are in place and are necessary to augment regional health research capability. Regional commitment and strong advocacy are required to strengthen policies on health research programmes aimed to provide evidence to justify health actions and practice. As much as these policies are required, they nonetheless have to be flexible and responsive to the short- and long-term national needs. Cooperation and active interaction among countries, not only in North–South alignments but also, and importantly, in South–South alliances and other possible configurations, will facilitate the development of clear policies in the countries and institutions that will enhance research capacity building and networking towards equitable health development. In this respect, African countries could put in place research-friendly legislative reforms that will facilitate exchange of expertise and the sharing of valuable epidemiological databases whilst ensuring intellectual property rights protection [6], [25]–[27]. Finally, strong and long-standing advocacy is needed to encourage governments and policy makers to extend more financial and political will towards the more diffuse and longer-term activities of scientific and health research, instead of the agendas of powerful lobbies and short-term economic targets. This would eventually lead to DECs building their own capacity to develop their appropriate enabling technologies and innovative products. Box 4 lists five recommendations for improving capacity in health research in general and helminth research in particular stemming from this report.

**Box 4. Five Recommendations for Improving Capacity in Helminthiasis Research in Disease-Endemic Countries (DECs)**

1. In addition to North–South collaborations, capacity building initiatives should also encourage South–South networking and fund schemes centred in DECs. Groups of research excellence in the South may then act as national and regional poles of reference and training
2. The initiatives above should have a formal mentorship component, with appointment of senior tutors from both within and outside DECs and the accompanying funding to facilitate mentoring meetings and exchanges with junior researchers
3. DECs may have reasonable capacity in some niche research areas, and in the case of helminthiases there is local strength in parasitology and entomology. Areas identified that require further capacity building to strengthen helminthiasis control are:
  1. Parasite and vector population biology and ecology
  2. Parasite and vector population genetics
  3. Mathematical and statistical analysis of transmission dynamics
  4. Epidemiological modelling of helminth infections
  5. Parasite and vector genomics, functional genomics and others (transcriptomics, proteomics), bioinformatics, and computational biology
  6. Detection and monitoring of resistance to anti-parasitic and anti-vectorial measures
  7. Optimisation of existing, and development of novel diagnostics, drugs, and vaccines
  8. Economic evaluation of the health impact of helminthiases and of deployed interventions for cost-effectiveness analysis of control programmes
4. In order to increase DEC research output, gain external visibility, and improve success rates in obtaining studentships, fellowships, and research awards in an increasingly competitive environment, capacity building strategies should include formal training in paper-writing, oral presentation skills, and grantsmanship
5. Monitoring and evaluation (M&E) instruments should be devised and deployed to assess the effectiveness of research capacity building strategies in the same rigorous way they are applied to quantify the impact of control interventions. This would provide evidence-based arguments for the continuation and improvement of capacity building

## Supporting Information

Figure S1 Concept and Strategy of the Hashimoto Initiative for Global Parasite Control. WB: World Bank; WHO: World Health Organization; MFA: Japan Ministry of Foreign Affairs; MHW: Japan Ministry of Health and Welfare; JICA: Japan International Cooperation Agency; G8: The Group of Eight (Canada, France, Germany, Italy, Japan, Russia, UK, USA); NMIMR: Noguchi Memorial Institute for Medical Research; WACIPAC: West Africa Centre for International Parasite Control; KEMRI: Kenya Medical Research Institute; ESACIPAC: Eastern and Southern Africa Centre of International Parasite Control; ACIPAC: Asian Centre of International Parasite Control (adapted from reference [1] of Text S1).
(PDF)

Table S1 Examples of Current Research Capacity Building Initiatives in the Area of Health Research, Helminthiases, and other Infectious Diseases of Poverty with Particular Reference to Africa.
(PDF)

Text S1 Examples of North–South and South–South Research and Capacity Building Initiatives in Helminthiases and other Infectious Diseases of Poverty.
(PDF)
