# Supplementary material for: A Research Agenda for Helminth Diseases of Humans: Health Research and Capacity Building in Disease-Endemic Countries for Helminthiases Control
Source: PLoS Negl Trop Dis. 2012 Apr 24;6(4):e1602. doi: 10.1371/journal.pntd.0001602 (PMC3335878; doi:10.1371/journal.pntd.0001602)
Supplement: Figure S1 — Concept and Strategy of the Hashimoto Initiative for Global Parasite Control. WB: World Bank; WHO: World Health Organization; MFA: Japan Ministry of Foreign Affairs; MHW: Japan Ministry of Health and Welfare; JICA: Japan International Cooperation Agency; G8: The Group of Eight (Canada, France, Germany, Italy, Japan, Russia, UK, USA); NMIMR: Noguchi Memorial Institute for Medical Research; WACIPAC: West Africa Centre for International Parasite Control; KEMRI: Kenya Medical Research Institute; ESACIPAC: Eastern and Southern Africa Centre of International Parasite Control; ACIPAC: Asian Centre of International Parasite Control (adapted from reference [1] of Text S1). (PDF) [file pntd.0001602.s001.pdf]

Mike Y. Osei-Atweneboana, Sara Lustigman, Roger K. Prichard, Boakye A. Boatin, María-Gloria Basáñez

A Research Agenda for Helminth Diseases of Humans: Health Research and Capacity Building in Disease-Endemic Countries for Helminthiases Control

Figure S1. Concept and Strategy of the Hashimoto Initiative for Global Parasite Control

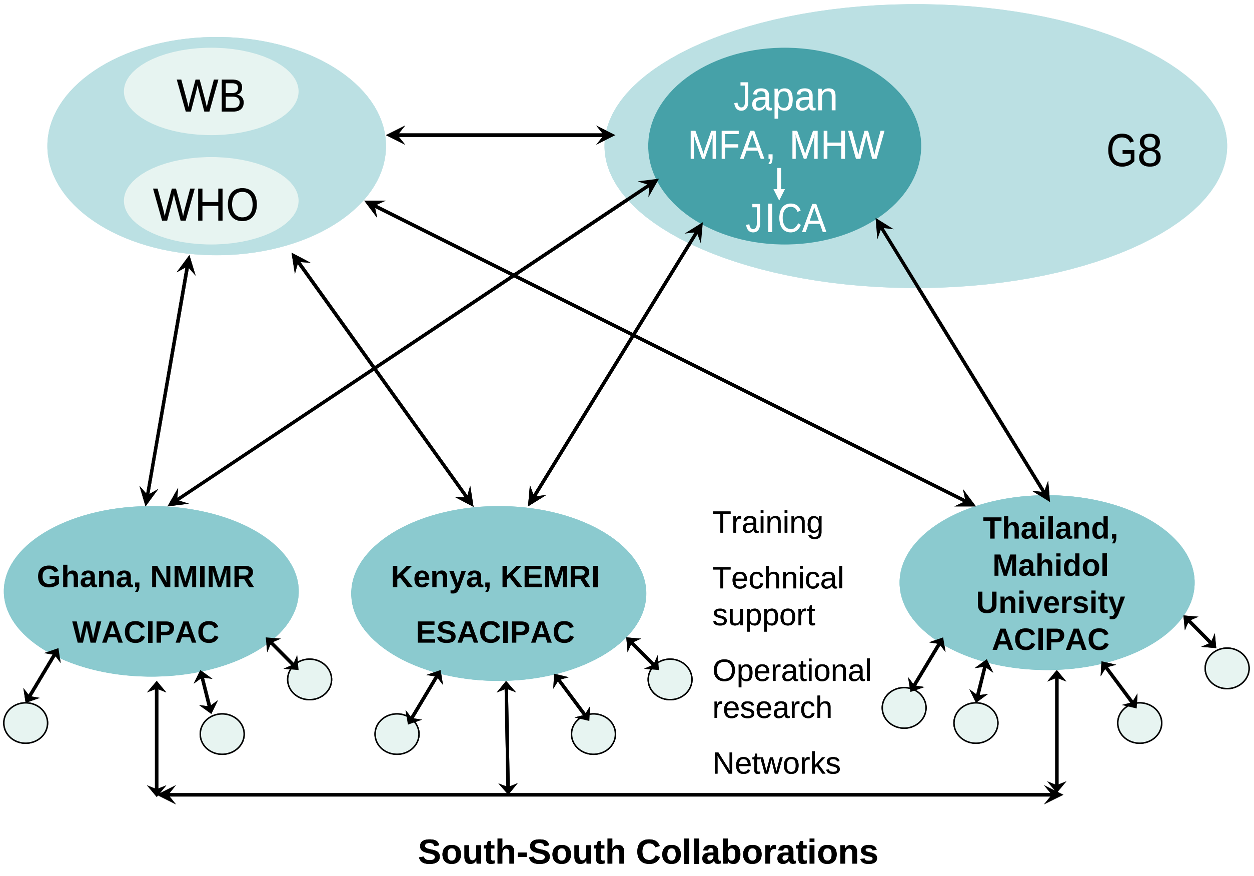

WB, World Bank; **WHO,** World Health Organization; **MFA,** Japan Ministry of Foreign Affairs; **MHW,** Japan Ministry of Health and Welfare; **JICA,** Japan International Cooperation Agency; **G8,** The Group of Eight (Canada, France, Germany, Italy, Japan, Russia, UK, USA); **NMIMR,** Noguchi Memorial Institute for Medical Research; **WACIPAC,** West Africa Centre for International Parasite Control; **KEMRI,** Kenya Medical Research Institute; **ESACIPAC,** Eastern and Southern Africa Centre of International Parasite Control; **ACIPAC,** Asian Centre of International Parasite Control (adapted from Ref [1] of Text S1).
