# Supplementary material for: A Research Agenda for Helminth Diseases of Humans: Health Research and Capacity Building in Disease-Endemic Countries for Helminthiases Control
Source: PLoS Negl Trop Dis. 2012 Apr 24;6(4):e1602. doi: 10.1371/journal.pntd.0001602 (PMC3335878; doi:10.1371/journal.pntd.0001602)
Supplement: Table S1 — Examples of Current Research Capacity Building Initiatives in the Area of Health Research, Helminthiases, and other Infectious Diseases of Poverty with Particular Reference to Africa. (PDF) [file pntd.0001602.s002.pdf]

Table S1. Examples of Current Research Capacity Building Initiatives in the Area of Health Research, Helminthiases and other Infectious Diseases of Poverty with Particular Reference to Africa

**Organization, country of origin**	**Initiative, type of support**	**Objectives**	**Link**
African Development Bank, UNAIDS, UNDP, UNFPA, UNICEF, WHO, World Bank	Harmonization for Health in Africa	To provide technical support and capacity building assistance to African countries; to work with existing development and financing frameworks towards preparation of poverty reduction strategy papers and other national development frameworks drawing on a country-led and participatory approach to health policies and programmes, including the achievement of the MDGs	http://www.eldis.org/go/topics/resource-guides/health-systems/key-issues/aid-architecture-in-health/moving-forward/-harmonization-for-health-in-africa-initiative
Australian Government, AusAID, Australia	Australia Awards (Scholarships) in Africa program<br>Specialised support through the Australia–Africa Partnership Facility	To assist African countries reach their MDGs in sectors where Australia has expertise and experience, including maternal and child health, and water and sanitation<br>To build Africa's human resource capacity	http://www.ausaid.gov.au/country/country.cfm?CountryID=95994596&Region=AfricaMiddleEast
Centre for Development Research, University of Bonn, Germany	Ghanaian-German Centre of Excellence for Development Studies and Health Research<br>Capacity development partnerships, PhD training	To establish centres of excellence at leading African universities and modern educational capacities with supra-regional influence. To expand the research capacity at these world-class hubs enabling the next generation of leaders to acquire a training in line with international standards	http://www.zef.de/1634.html<br>http://www.isser.edu.gh/
Calouste Gulbenkian Foundation, Portugal	Doctoral Fellowship Programme in NTDs for nationals of Portuguese-speaking African countries	To foster research in the area of Neglected Tropical Diseases in Portuguese-speaking African countries	http://www.ntd-africa.net/
Centre for Neglected Tropical Diseases (CNTD), Liverpool School of Tropical Medicine (LSTM), UK	Doctoral Fellowship Programme in lymphatic filariasis (LF) for nationals of LF-endemic countries in the Africa and Asia-Pacific regions	To develop skills to work in new and promising areas of research targeted at addressing key gaps in knowledge regarding the most effective means for achieving elimination of lymphatic filariasis by 2020	http://www.ntd-africa.net/

Fogarty International, NIH, USA	Global Infectious Disease Research Training Program (GID)<br>Master's or Doctoral degrees and other training taking ≥6 months<br>Training and/or mentoring taking 3–6 months<br>Advanced in-country mentored research undertaken by the trainee in his/her home country upon completion of his/her initial period of long-term training under the program	To address research training needs related to infectious diseases that are predominantly endemic in or impact upon people living in developing countries<br>To build a critical mass of researchers and support staff to conduct independent infectious disease research in developing country institutions<br>Training areas include specialized clinical, laboratory, research or administrative/ business skills; research methodology, research ethics and compliance issues, programme and grants administration and financial management, grant writing, preparation of scientific manuscripts, data management, informatics, and other relevant areas	http://www.fic.nih.gov/Programs/Pages/infectious-disease.aspx
International Development Research Centre (IDRC), Canada	Research support led by developing country principal investigator	To support development-orientated research and capacity strengthening in developing countries, including in the area of health	http://publicwebsite.idrc.ca/EN/Programs/Health_and_Health_Systems/Pages/default.aspx
Japan International Cooperation Agency (JICA), Japan	Science and Technology Research Partnership for Sustainable Development	To gain new knowledge that leads to solutions for global issues such as environmental and climate change, infectious diseases, water and disasters, and to make a concrete contribution to society through the results of the research<br>To strengthen health administration functions at the state or regional/district level in nations promoting decentralization, such as Tanzania	http://www.jica.go.jp/english/operations/thematic_issues/health/activity.html
	Strengthening of Mathematics and Science Education – Western, Eastern, Central and Southern Africa (SMASE-WECSA)	To improve mathematics and science education across Africa	http://www.jica.go.jp/english/operations/thematic_issues/south/project05.html
Leverhulme Trust; Royal Society, UK	Leverhulme-Royal Society Africa Award<br>3-year project grants	To help develop and maintain excellence in science in Ghana and Tanzania; to strengthen research and training capacity of the African institution	http://royalsociety.org/grants/schemes/leverhulme-africa/

Makerere University, Uganda (Leader); University of Cambridge and London School of Hygiene and Tropical Medicine, UK provide co-supervision. Funded by the Wellcome Trust African Institutions Initiative	Training Health Researchers into Vocational Excellence in East Africa (THRiVE) Partnership<br><br>PhD and post-doctoral fellow-ships; registered in the African partner universities. First fellowships have begun in 2011	To strengthen institutional research capacity in East Africa, and support the next generation of East African researchers to become internationally competitive and self-sustaining scientific leaders, seeding a regional research community with the critical mass to address African health priorities	http://www.thrive.cam.ac.uk/
Medical Research Council (MRC) and Department for International Development (DFID), UK	MRC/DFID African Research Leader scheme<br><br>5-year project grants, renewable	To strengthen research leadership and capacity across sub-Saharan Africa by attracting and retaining researchers of high ability	http://www.mrc.ac.uk/Fundingopportunities/Calls/AfricanResearchLeader/MRC006652#P67_7656
National Research Council (CNPq), Brazil	Pro-Africa Program for Thematic Cooperation in Science and Technology<br><br>Air tickets and accommodation expenses for researchers, doctorate students and African experts carrying out joint science, technology and innovation projects in Brazil<br><br>Exploratory visits throughout the country	To promote the of field of international cooperation through bilateral agreements and multinational programmes, giving support to research, development and innovation projects<br><br>Partners are Cape Verde, Mozambique and other African countries	http://www.brasil.gov.br/sobre/education/access-to-university/cnpq
National Science Foundation (NSF), USA	DIMACS/MBI US–African BioMathematics Initiative<br><br>Epidemiological data modelling clinics; advanced study institutes and workshops; visiting researcher programs	To identify key biomathematical challenges arising from problems of Africa, creating long-lasting partnerships between US and African mathematical scientists, and training junior researchers to work in the field of biomathematics (including health research and epidemiological modelling)	http://dimacs.rutgers.edu/US-AfricanInitiative/

Noguchi Memorial Institute for Medical Research (NMIMR), University of Ghana, Ghana, with funding from the B&MGF	Postdoctoral Fellowship Program of Research for Control of Poverty-Related Diseases<br><br>2-year postdoctoral fellowship, renewable to 3 years	To train young African scientists toward building a critical mass for control of poverty-related diseases, equip them to compete effectively for international funding for research work in Africa, offer them international exposure, and facilitate their networking and collaboration with other institutions	http://www.noguchimedres.org/
US Agency for International Development (USAID), USA	Neglected Tropical Diseases (NTD) Program<br><br>Integrated NTD control in Burkina Faso, Ghana, Mali, Niger, Sierra Leone, Southern Sudan, and Uganda	To make a large-scale, cost-effective contribution to the global effort to reduce the economic and epidemiological burden of NTDs<br><br>To provide financial and technical support to DECs to deliver regular, large-scale treatment for populations at risk of helminthiases and trachoma	http://www.neglecteddiseases.gov/index.html
Volkswagen Foundation, Germany; Gulbenkian Foundation, Portugal; Fondation Mérieux, France; Nuffield Foundation, UK; Fondazione Cariplo, Italy	European Foundation Initiative for African Research into Neglected Tropical Diseases (EFINTD)<br><br>3-year fellowships	To enable African scientists to enter an academic career and develop their own projects on neglected tropical diseases in their countries of origin.<br><br>This initiative started in 2008, funding collaborative research between European senior scientists and African young scientists, with the African scientists driving the research supported by the European partners	http://www.ntd-africa.net/
Wellcome Trust, UK	African Institutions Initiative Consortia (e.g. THriVE)	To fill the gap between Africa's pressing health problems and the deficit of scientists to tackle them	http://www.wellcome.ac.uk/Funding/Biomedical-science/Funding-schemes/Strategic-awards-and-initiatives/WTD028338.htm

Abbreviations: AusAID, Australian Agency for International Development; **B&MGF,** Bill and Melinda Gates Foundation; **CNPq,** National Research Council of Brazil; **CNTD,** Centre for Neglected Tropical Diseases (Liverpool, UK); **DEC,** Disease Endemic Country; **DFID,** Department for International Development, UK; **DIMACS/MBI,** Center for Discrete Mathematics and Theoretical Computer Science/US–African Biomathematics Initiative; **EFINTD,** European Foundation Initiative for African Research into Neglected Tropical Diseases; **GID,** Global Infectious Disease Research Training Program, USA; **IDRC,** International Development Research Centre, Canada; **JICA,** Japan International Cooperation Agency; **LSTM,** Liverpool School of Tropical Medicine; **MDG,** Millennium Development Goals; **MRC,** Medical Research Council, UK; **NIH,** National Institutes of Health, USA; **NMIMR,** Noguchi Memorial Institute for Medical Research; **NSF,** National Science Foundation, USA; **NTD,** Neglected Tropical Disease; **SMASE-WECSA,** Strengthening Mathematics and Science Education in Western, Eastern, Central, and Southern Africa; **THRiVE,** Training Health Researchers into Vocational Excellence in East Africa; **UNAIDS,** United Nations Programme on HIV/AIDS; **UNDP,** United Nations Development Programme; **UNFPA,** United Nations Population Fund; **UNICEF,** United Nations Children's Fund; **USAID,** United States Agency for International Development.
