# Supplementary material for: A Research Agenda for Helminth Diseases of Humans: Health Research and Capacity Building in Disease-Endemic Countries for Helminthiases Control
Source: PLoS Negl Trop Dis. 2012 Apr 24;6(4):e1602. doi: 10.1371/journal.pntd.0001602 (PMC3335878; doi:10.1371/journal.pntd.0001602)
Supplement: Text S1 — Examples of North–South and South–South Research and Capacity Building Initiatives in Helminthiases and other Infectious Diseases of Poverty. (PDF) [file pntd.0001602.s003.pdf]

Supplementary Text S1. Examples of North–South and South–South Research and Capacity Building Initiatives in Helminthiases and Other Infectious Diseases of Poverty

Hashimoto Initiative. The Japan International Cooperation Agency (JICA), created in 1974, and the Hashimoto Initiative, launched in 1998 [1] started the Global Parasite Control Initiative, establishing disease reference centres in endemic countries (one in Asia and two in Africa) which are responsible for training and coordination of regional activities for the control and prevention of parasitic diseases. These centres are located at Mahidol University in Thailand (the Asian Centre for International Parasite Control or ACIPAC, inaugurated in 2000); the Kenya Medical Research Institute (KEMRI) in Nairobi (the Eastern and Southern Africa Centre of International Parasite Control or ESACIPAC, established in 2001); and the Noguchi Memorial Institute for Medical Research (NMIMR) in Legon, Ghana (the West African Centre for International Parasite Control or WACIPAC, founded in 2004). Figure S1 illustrates the flow diagram and structure of the partnership and the resulting South–South research capacity building networks that ensue [2].

Partnership for Child Development and Schistosomiasis Control Initiative. The Partnership for Child Development (PCD), hosted at Imperial College London, and created in 1992 (http://www.child-development.org/Pages/default.aspx) focuses on programmatic capacity building of school health and nutrition interventions, including deworming [3–5]. The Schistosomiasis Control Initiative (SCI, described more extensively in [6]) was established in 2002, also at Imperial College [7]. It has assisted eight African countries (Burundi, Burkina Faso, Mali, Niger, Rwanda, Tanzania (including Zanzibar), Uganda and Zambia) to establish nation-wide control of schistosomiasis and soil-transmitted intestinal helminths (STHs). In each country, the most heavily infected regions have been identified; local health staff and

teachers have been trained, and health education has been provided to the people in those regions; and the drugs praziquantel and albendazole have been distributed to treat against schistosomiasis and intestinal helminths respectively. As the programmes have expanded, their impact has been monitored in each country to demonstrate treatment effects on parasite abundance, transmission and morbidity [8–11].

The SCI has contributed to the development of local capacity for sustained implementation of schistosomiasis and helminth control programmes. Local and international partnerships have been forged to improve training and treatment delivery, and to assist other African nations in the development of national control plans and research programmes. Cameroon, Kenya, Malawi, and Mozambique have benefited in one way or another from small SCI grants (http://www1.imperial.ac.uk/publichealth/departments/ide/groupsandcollabs/thesci/).

Regional Network for Asian Schistosomiasis and Other Zoonotic Helminths (RNAS+). This network, sponsored by the Special Programme for Research and Training in Tropical Diseases of the World Health Organization, TDR, aims to strengthen communication, cooperation and coordination among scientists and control authorities concerned with schistosomiasis japonica and, since 2007, all zoonotic helminthic infections in the Southeast Asian region (http://www.rnas.org.cn). The RNAS+ comprises five member countries (Cambodia, Indonesia, Lao People's Democratic Republic, People’s Republic of China, and the Philippines) for coordination of research, control and surveillance of schistosomiasis and other zoonotic helminthiases, dissemination of information about ongoing research and training activities, and development of standardized protocols for infection and disease surveillance [12–14].

Regional Network for Schistosomiasis in Africa (RNSA). This network, also supported by the TDR, brings together researchers and control personnel working on schistosomiasis in sub-Saharan Africa to share results, experiences, and capacity building. The aim of RNSA is to provide a platform to improve the quality of schistosomiasis operational research relevant to effective control of schistosomiasis in Africa, facilitate the transformation of research findings into practical application, and influence national and regional decisions concerning schistosomiasis research and control in Africa (http://www.rnsa.org.zm). The RNSA has established South–North partnerships with the SCI and the Danish Bilaharziasis Laboratory (DBL)-Institute for Health Research and Development, as well as South–South links with ESACIPAC, among others. In 2010, another South–South partnership, with RNAS+, was established with sponsorship by the TDR as described in the main text.

Lymphatic Filariasis Support Centres (LFSCs). The LFSCs, created in 2000 at the Liverpool School of Tropical Medicine and since 2008 incorporated into the Centre for Neglected Tropical Diseases, Liverpool, UK (http://www.cntd.org/); the Task Force for Global Health in Georgia, Atlanta, USA (http://www.taskforce.org/our-work/programs/lymphatic-filariasis-support-center), and more recently, in 2007, at the NMIMR, University of Ghana at Legon, Accra, Ghana (http://98.130.228.222/LF_center.php), are involved in capacity building from doctoral fellowships (Table 1) to providing technical expertise, strengthening laboratories in disease-endemic countries (DEC) (e.g. in Egypt, Kenya, Malawi, Ghana, Sierra Leone, and Sri Lanka), and collaborating closely (locally, nationally, and internationally) with partners involved in global health and the elimination of lymphatic filariasis worldwide [15].

Medical Research Council Laboratories (MRC) in The Gambia. Addressing other infectious diseases of poverty, additional partnerships involve the MRC Laboratories in The Gambia, created in 1947, with a staff that is more than 80% Gambian. Its work focuses on tuberculosis, acute respiratory infections, viral diseases, and malaria. Close collaborations exist with local non-governmental organizations and with UK-based and international groups. The UK Department for International Development (DFID) is a crucial partner, as are the World Health Organization (WHO), the United Nations Global Fund, the Gates Malaria Partnership, the European Commission, and a variety of academic bodies and institutions (http://www.mrc.ac.uk/Ourresearch/Unitscentresinstitutes/Profiles/TheGambia/index.htm). Emphasis has been placed on developing a professional training pathway in biomedical sciences. For instance, the MRC-Westminster University Diploma in Biochemical Sciences, set up in 2001, has had its third batch of students graduating in January 2007. Gambian laboratory technicians have the opportunity to complete an in-house course and receive the 'MRC Certificate in Biomedical Sciences'. In addition, Gambian staff is enrolled in distance learning through the University of South Africa, while Gambian and international students have the opportunity of completing their PhD programmes at the unit.

NIAID/NIH International Centers of Excellence for Malaria Research. To address capacity building in malaria, and to accelerate the control and elimination of malaria, the National Institute of Allergy and Infectious Diseases (NIAID), part of the National Institutes of Health (NIH) of the USA, has established 10 malaria research centres around the world through 7-year awards that will support International Centers of Excellence for Malaria Research (ICEMRs) in regions where malaria is endemic, including parts of Africa (Malawi, Mali, southern Africa (including Zambia, Zimbabwe and South Africa), and Uganda); India; Latin America (Colombia and Peruvian/Brazilian Amazonia); the Pacific Islands (Papua New Guinea, Solomon Islands, and Vanuatu), and Southeast Asia (including China, Myanmar,

and Thailand). These centres will focus on malaria research (including the study of co-infection with other parasites and pathogens whenever pertinent and appropriate), and will also offer training, career development, and capacity building locally and regionally (http://www.niaid.nih.gov/LabsAndResources/resources/icemr/Pages/centers.aspx).

Ghanaian-German Centre of Excellence for Development Studies. This is a collaborative venture between the Institute of Statistical, Social and Economic Research (ISSER), University of Ghana, and the Centre for Development Research (ZEF), University of Bonn, Germany. The Centre is funded by the German Academic Exchange Service (DAAD) under the Centres of Excellence for Teaching and Research to Train Future Leaders in sub-Saharan Africa Initiative (http://www.isser.edu.gh/ and http://www.zef.de).

Kumasi Centre for Collaborative Research (KCCR). The KCCR is a joint venture between the Ministry of Health of the Republic of Ghana, the Kwame Nkrumah University of Science and Technology (KNUST) in Kumasi, Ghana, and the Bernhard Nocht Institute for Tropical Medicine (BNITM), Hamburg, Germany. The KCCR is committed to establishing a first class research institution for tropical medicine in Ghana, combining research, capacity building, and educational programmes (http://kccr-ghana.org/index.php) with other Ghanaian organizations and academic bodies in Germany (Universities in Berlin, Bonn, Hamburg, and Munich), the UK (St. Georges Hospital Medical School, London), and the USA (University of Alabama at Birmingham). Capacity building and research on neglected tropical diseases (NTDs) and helminthiases, has involved KCCR in conducting clinical trials and research of anti-wolbachial therapies (detailed in [16]) for the control of infection and morbidity in human onchocerciasis and lymphatic filariasis [17,18].

Research Institute for Development (Institut de Recherche pour le Développement, IRD). IRD manages an extensive research and development portfolio with emphasis on questions pertinent to the 'South', for the 'South' and with the 'South' (http://www.ird.fr/), and in this respect it is different and unique in the European research funding landscape. In addition to tropical, neglected, and emerging diseases, IRD focuses on important current problems such as climate change, biodiversity, water and the environment, poverty reduction, and population migration, among others, with active scientific programmes in Africa, the Asia-Pacific region, Latin America and the Caribbean, and the Mediterranean region. IRD is under the joint authority of French organisations responsible for international development assistance on the one hand, and scientific research on the other, and its three main functional areas are research, advisory services, and capacity building. Its particular mandate is to build capacity and foster innovation in the scientific communities of the South.

Latin American Network for Research on Bioactive Natural Compounds (LANBIO). An excellent example of South–South partnership is the Latin American Network for Research on Bioactive Natural Compounds (LANBIO, http://abulafia.ciencias.uchile.cl/lanbio/), which was created in 1991 to promote research on natural products in the region with potential use in the pharmaceutical (and agro-chemical) industry [19]. This partnership also represents an important avenue for promoting and strengthening local research capacity, generating new knowledge as well as attracting resources for sustainable research activities, all of which are leading to the production and testing of candidate products to meet current international quality standards. LANBIO has been pivotal in providing highly specialized professional training, promoting multicentre and multinational basic as well as clinical and epidemiological research, and incorporating new technologies and procedures.

South–South Initiative for Infectious Diseases of Poverty (SSI). Another significant development is the South–South Initiative for Infectious Diseases of Poverty (SSI), initiated in 2001 with support of the Pathogenesis and Applied Genomics Committee of TDR, and now entering its second phase of operation. The main goal of the initiative is to promote interaction and research collaboration between researchers interested in infectious diseases of poverty in DECs across Africa, Asia, and Latin America, facilitating the sharing of resources among research groups in these regions, enhancing and optimizing scientific opportunities; and helping to address health disparities. The SSI is managed by a coordinating committee representing African, Asian, and Latin American countries. Such South–South collaborations will help the least-developed countries, either through the manufacture and export of low-cost products or through technical assistance and more specific, capacity building (http://www.ssi-tdr.net).

African Network for Drugs and Diagnostics Innovation (ANDI). Launched in Abuja, Nigeria, in 2008 by the WHO/TDR, ANDI is now fully supported by African governments (http://www.andi-africa.org/). The aim of ANDI is to promote and sustain African-led health product innovation, which are specifically aimed to address African public health needs and to enhance research capacity building.

New Partnership for Africa's Development (NEPAD). Such African collaborations are likely to promote much of the necessary progress, especially with the establishment of the New Partnership for Africa's Development (NEPAD), adopted in Lusaka, Zambia in 2001. NEPAD is a radically new intervention, spearheaded by African leaders to pursue new priorities and approaches to the political and socio-economic transformation of Africa through the development of a technical body of the African Union. Its objective is to enhance Africa's growth, development and participation in the global economy, facilitating

partnerships within and between African countries, and with the international community [20,21]. In 2011, NEPAD marked its 10th anniversary with, among other events, a high-level colloquium of African scholars and all-stakeholders forum to reflect on the way forward. One of NEPAD's primary objectives is to work towards greater economic growth and poverty eradication on the African continent through linking together key areas of human development, including education, science and technology, and healthcare towards achieving the millennium development goals (MDGs) (http://www.nepad.org).

Congress of African Scientists and Policy Makers (CASP). Even though the countries of the African Union had committed to invest 1% of their GDP in science and technology activities [22], most countries have not yet met this target. In the first Congress of African Scientists and Policy Makers (CASP), in October, 2006 in Alexandria, Egypt, it was concluded that human capital development depended on the quality of science education, increasing the number of science students (male and female), enhancing science and technology research collaboration and networking, and engaging Africa's centres of excellence.

References

1. Crompton DWT, Montresor A, Nesheim MC, Savioli L (eds) (2003) Controlling disease due to helminth infections. Geneva: World Health Organization. http://www.who.int/wormcontrol/documents/en/Controlling%20Helminths.pdf

2. Takeuchi T, Kasai T, Hara T (2003) The Hashimoto Initiative and the control of soil-transmitted helminth infections: the Japanese challenge. In Crompton DWT, Montresor A, Nesheim MC, Savioli L (eds). Controlling disease due to helminth infections. Geneva: World Health Organization, pp. 81–85.

3. Satoto, Hendratno S, Subagyo H, Rafilluddin Z, Mogensen M et al. (2003) Partnership for Child Development: an international programme to improve the health of school-age children by school-based health services including deworming. In Crompton DWT, Montresor A, Nesheim MC, Savioli L (eds). Controlling disease due to helminth infections. Geneva: World Health Organization, pp. 93–97.

4. Partnership for Child Development (2008) Annual Report. http://www.child-development.org/Lists/PCD%20Publications/Attachments/53/PCD%20ANNUAL%20REPORT%2008%20web.pdf

5. Partnership for Child Development (2009) Annual Report. http://www.child-development.org/Lists/PCD%20Publications/Attachments/55/PCD%20ANNUAL%20REPORT%20LOW.pdf

6. Boatin BA, Basáñez MG, Prichard RK, Awadzi K, Barakat RM, et al. (2012). A research agenda for helminth diseases of humans: towards control and elimination. PLoS Negl Trop Dis (accepted).

7. Fenwick A, Webster JP, Bosqué-Oliva E, Blair L, Fleming FM, et al. (2009) The Schistosomiasis Control Initiative (SCI): rationale, development and implementation from 2002–2008. Parasitology 136: 1719–1730.

8. Kabatereine NB, Brooker S, Koukounari A, Kazibwe F, Tukahebwa EM, et al. (2007) Impact of a national helminth control programme on infection and morbidity in Ugandan schoolchildren. Bull World Health Organ 85: 91–99.

9. Koukounari A, Gabrielli AF, Touré S, Bosqué-Oliva E, Zhang Y, et al. (2007) *Schistosoma haematobium* infection and morbidity before and after large-scale administration of praziquantel in Burkina Faso. J Infect Dis 196: 659–669.

10. Zhang Y, Koukounari A, Kabatereine N, Fleming F, Kazibwe F, et al. (2007) Parasitological impact of 2-year preventive chemotherapy on schistosomiasis and soil-transmitted helminthiasis in Uganda. BMC Med 5: 27.

11. French MD, Churcher TS, Gambhir M, Fenwick A, Webster JP, et al. (2010) Observed reductions in *Schistosoma mansoni* transmission from large-scale administration of praziquantel in Uganda: a mathematical modelling study. PLoS Negl Trop Dis 4: e897.

12. Zhou X-N, Ohta N, Utzinger J, Bergquist R, Olveda RM (2008) RNAS(+): a win-win collaboration to combat neglected tropical diseases in Southeast Asia. Parasitol Int 57: 243-245.

13. Zhou X-N, Bergquist R, Olveda R, Utzinger J (eds.) (2010a) Important helminth infections in Southeast Asia. Diversity, potential for control and prospects for elimination, Part A. Adv Parasitol 72: 1–455.

14. Zhou X-N, Bergquist R, Olveda R, Utzinger J (eds.) (2010b) Important helminth infections in Southeast Asia. Diversity, potential for control and prospects for elimination, Part B. Adv Parasitol 73: 1–462.

15. Addiss D (2010) The Global Alliance to Eliminate Lymphatic Filariasis. The 6th Meeting of the Global Alliance to Eliminate Lymphatic Filariasis: a half-time review of lymphatic filariasis elimination and its integration with the control of other neglected tropical diseases. Parasit Vectors 3: 100.

16. Prichard RK, Basáñez MG, Boatin BA, McCarthy J, García HH, et al. (2012) A research agenda for helminth diseases of humans: intervention for control and elimination. PLoS Negl Trop Dis (this issue).

17. Taylor MJ, Awadzi K, Basáñez MG, Biritwum N, Boakye D, et al. (2009) Onchocerciasis Control: vision for the future from a Ghanaian perspective. *Parasit Vectors* 2: 7.

18. Debrah AY, Mand S, Marfo-Debrekyei Y, Batsa L, Albers A, et al. (2011) Macrofilaricidal activity in *Wuchereria bancrofti* after 2 weeks treatment with a combination of rifampicin plus doxycycline. *J Parasitol Res* 2011, 201617.

19. Dujardin JC, Herrera S, do Rosario V, Arevalo J, Boelaert M, et al. (2010) Research priorities for neglected infectious diseases in Latin America and the Caribbean region. PLoS Negl Trop Dis 4: e780.

20. Volmink J, Dare L (2005) Editorial: Addressing inequalities in research capacity in Africa.BMJ 331: 705–706.

21. Nkuhlu WL (2005) The new partnership for Africa's development: the journey so far. http://www.africarecruit.com/downloads/NEPAD-JOURNEYSOFAR.pdf

22. CASP (2006) First African Union Congress of Scientists and Policy Makers. First Ordinary Session, Alexandria, Egypt, October 27-29, 2006. http://www.unesco.org/new/fileadmin/MULTIMEDIA/HQ/SC/pdf/sc_1st_african_congress_en.pdf
